# A Systematic Review of Keratinocyte Secretions: A Regenerative Perspective

**DOI:** 10.3390/ijms23147934

**Published:** 2022-07-19

**Authors:** Ahmed T. El-Serafi, Ibrahim El-Serafi, Ingrid Steinvall, Folke Sjöberg, Moustafa Elmasry

**Affiliations:** 1 Department of Biomedical and Clinical Sciences, Linköping University, 58183 Linkoping, Sweden; ingrid.steinvall@regionostergotland.se (I.S.); folke.sjoberg@liu.se (F.S.); moustafa.elmasry@liu.se (M.E.); 2 Department of Hand Surgery, Plastic Surgery and Burns, Linköping University, 58183 Linkoping, Sweden; i.elserafi@ajman.ac.ae; 3 Basic Medical Sciences Department, College of Medicine, Ajman University, Ajman P.O. Box 346, United Arab Emirates

**Keywords:** keratinocyte, keratinocyte secretion, skin regeneration, inflammatory mediator, stem cell differentiation, growth factor

## Abstract

Cell regenerative therapy is a modern solution for difficult-to-heal wounds. Keratinocytes, the most common cell type in the skin, are difficult to obtain without the creation of another wound. Stem cell differentiation towards keratinocytes is a challenging process, and it is difficult to reproduce in chemically defined media. Nevertheless, a co-culture of keratinocytes with stem cells usually achieves efficient differentiation. This systematic review aims to identify the secretions of normal human keratinocytes reported in the literature and correlate them with the differentiation process. An online search revealed 338 references, of which 100 met the selection criteria. A total of 80 different keratinocyte secretions were reported, which can be grouped mainly into cytokines, growth factors, and antimicrobial peptides. The growth-factor group mostly affects stem cell differentiation into keratinocytes, especially epidermal growth factor and members of the transforming growth factor family. Nevertheless, the reported secretions reflected the nature of the involved studies, as most of them focused on keratinocyte interaction with inflammation. This review highlights the secretory function of keratinocytes, as well as the need for intense investigation to characterize these secretions and evaluate their regenerative capacities.

## 1. Introduction

The skin is the largest organ in the body and provides protection against pathogens, as well as against mechanical, thermal, and physical injuries. Skin also acts as a sensory organ and helps regulate body temperature. In addition, it is essential for vitamin D production. Skin consists of three main layers: the epidermis, which is the outermost layer, the dermis, and the hypodermis. The epidermis has three main types of cells: keratinocytes (skin cells), melanocytes (pigment-producing cells), and Langerhans cells (immune cells). Keratinocytes are the main cell type in the epidermis and are therefore of interest in skin regeneration [1,2,3]. The dermis is the supporting layer that provides elasticity and houses the skin’s appendages. The hypodermis is the subcutaneous fat, which helps with skin mobility and provides a thermal insulation layer, as well as being a local source for mesenchymal stem cells. Physiological regeneration is a natural process that follows limited skin damage and depends on the general health of the patient, as well as the occurrence of wound infection. Difficult-to-heal wounds are those wounds which do not follow the normal sequence of healing. These wounds can be developed in patients with extensive skin-loss conditions, such as in burn victims, as well as following a sequence of chronic ulcers due to vascular, metabolic, iatrogenic, or idiopathic etiology [4]. The defective area is usually treated for infection, debrided, and covered with standard or advanced dressings to encourage natural healing mechanisms. The patient usually attends weekly hospital visits for follow-up and dressing changes. Surgery can be carried out on difficult-to-heal wounds, which can range from split-thickness or punch-biopsy skin grafts to skin flaps, and repeated surgeries may be required according to the wound response. The persistence of the wound may be complicated by local or systemic infection, which may require a battery of medications, including antibiotics. Moreover, severe cases may need hospitalization and even intensive care. With the failure of classical management methods, regenerative therapy represents a hope for those patients as a new approach to improve the clinical outcome. The traditional regenerative approach depends on obtaining a skin biopsy, isolating the keratinocytes, and expanding them in the lab with the aim of reapplication at the site of the skin defect. Unfortunately, keratinocytes are difficult to maintain and slow to grow in ex vivo culture, with huge variabilities in outcome among donors [5]. Furthermore, the clinical application of these cells is associated with several challenges. For example, the use of any animal-derived product should be avoided, including murine fibroblasts as a feeder layer, media additives, or fetal calf serum. These limitations favor the use of stem cells as a progenitor for epidermal cells. Stem cells can be obtained in abundance through lipoaspiration or bone marrow biopsy, and these cells can be expanded in culture with relative ease. However, the differentiation of stem cells into keratinocytes is a challenging process. Despite the presence of several published protocols, the outcome of differentiation is variable and inconsistent among different donors, both in our unpublished data, as well as in the literature [6]. Nevertheless, co-culturing stem cells with keratinocytes or applying conditioned keratinocyte media to stem cells remains the gold standard for the efficient differentiation of stem cells into keratinocytes [7,8]. This approach has been proven in other systems as well. For example, the secretions released by mesenchymal stem cells in a co-culture system with neuroretinal explants protected the latter from degenerative changes through the upregulation of several neurotropic factors [9]. In physiological conditions, cells communicate with each other through the release of certain proteins, messenger RNA, and micro-RNA. The proteins can include locally acting growth factors, transcription factors, hormones, and inflammatory mediators. The latter can be markedly upregulated in response to cellular stress, such as infection or hypersensitivity reactions [10]. In vitro, the released product can be collected and characterized from the cell culture supernatant. The presence of these “extracellular messages” can trigger the differentiation of stem cells into the same cell-type that produced the secretions. However, the co-culture approach has not been accepted for the clinical differentiation of stem cells for technical reasons. Knowledge of the key inducers of differentiation is crucial for determining a chemical formula for keratinocyte differentiation, according to the European Medicines Agency’s guidelines [11]. Furthermore, detailed characterization of keratinocyte secretions may provide further understanding of the function of these cells and direct the management plans for several skin conditions. Accordingly, the objective of this systematic review is to recognize the most-reported keratinocyte secretions in the literature and identify those which are possibly related to stem cell differentiation.

## 2. Methods

**Figure 1:**
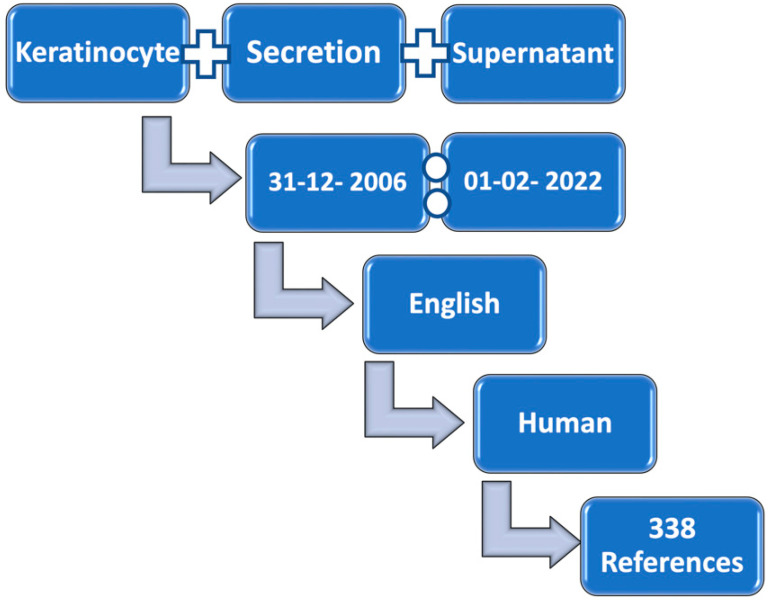
Flow chart of the online search strategy used in this study.

This study was based on a systematic review of the available data on keratinocyte secretions. For this purpose, studies published over the last 15 years were reviewed. As studying cell secretion in vivo is extremely difficult, the included studies investigated keratinocyte secretions in cell culture supernatant, under standard culturing conditions, and without the application of any inducer or abnormal challenge. The strategy of this study depended on a web-based search of the MEDLINE database for references in the life sciences and biomedicine, using the PubMed search engine, as well as Elsevier’s abstract and citation database, Scopus. The keywords for this search were (keratinocyte) AND (secretion) AND (human) AND (supernatant). The online search was performed for articles published between 31 December 2006 and 1 February 2021 and was limited to the English language (Figure 1). The references were individually reviewed by two of the authors. Initially, each record was checked for the presence of the keywords, and those that fulfilled the criteria were included in the analysis. There were no disagreements regarding inclusion/exclusion. In the case of disagreement, the articles would have been discussed until consensus was reached. The model considered keratinocytes or keratinocyte-like cell lines cultured in vitro when the culture supernatant was collected and analyzed for one or more of the secretory products. The secreted proteins were analyzed using the STRING database (https://string-db.org/, last accessed on 2 June 2022). The clustered data were investigated for enriched biological processes according to gene ontology (GO) terms.

## 3. Results and Discussion

The number of records retrieved using the search engine PubMed was 227, while Scopus retrieved 205 records. The overlap between the hits discovered by the 2 search engines was 94 records, which produced a total of 338 references. Interestingly, only 100 references could be included in the final analysis according to our inclusion criteria [12,13,14,15,16,17,18,19,20,21,22,23,24,25,26,27,28,29,30,31,32,33,34,35,36,37,38,39,40,41,42,43,44,45,46,47,48,49,50,51,52,53,54,55,56,57,58,59,60,61,62,63,64,65,66,67,68,69,70,71,72,73,74,75,76,77,78,79,80,81,82,83,84,85,86,87,88,89,90,91,92,93,94,95,96,97,98,99,100,101,102,103,104,105,106,107,108,109,110,111]. Most of these articles were based on the use of primarily isolated keratinocytes from human skin samples (58%), while 40% of the studies used the cell line HaCaT. The latter is a line of spontaneously transformed primary human keratinocytes, which is a commercially available and widely used model for normal epidermal cells. The rest of the studies (2%) used immortalized cell lines. Out of the included studies, 45% reported a single-cell excretory product, while 55% described several secretions. The absence of a detailed description of keratinocyte secretions under physiological conditions was the main reason for this study.

### 3.1. Stratification of the Retrieved Articles

**Figure 2:**
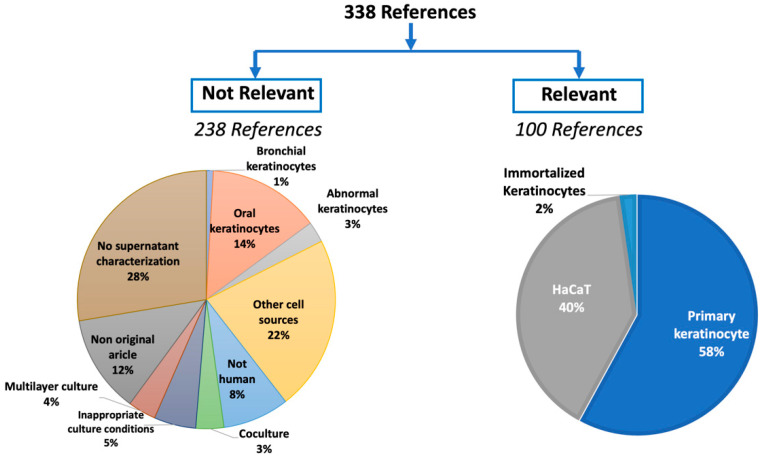
Stratification of the retrieved articles (338) into relevant (100) and irrelevant (238) references. The relevant references were further divided according to the study cell-type, while the irrelevant studies were divided according to the reason for rejection.

The retrieved references were individually reviewed by the research team, and 238 references were excluded for several reasons (Figure 2). The most common reason for exclusion (28%) was that the articles did not analyze the culture supernatant. In most of these articles, the supernatant isolated from other cell types or microorganisms was collected and applied to cultured keratinocytes. The keratinocyte secretions were not investigated because the focus was the metabolic and behavioral changes of the cells in response to the supernatant, mimicking certain pathological conditions. Hence, the objectives of these studies were beyond the scope of this review. Oral and bronchial keratinocytes were involved in 14% and 1% of the excluded articles, respectively. Studies involving primary cells, apart from epidermal-derived keratinocytes, were excluded. There are essential differences between keratinocytes according to their sources. For example, the rate of wound healing and the incidence of scarring are different between oral and epidermal keratinocytes as the healing rate and properties are more effective in the oral epithelium [112]. Furthermore, keratinocytes from the two sources differ in cell–cell interactions in terms of their expression of tight junctions, which increase epidermal keratinocyte permeability upon histamine stimulation [113]. Similarly, the secretory functions of epidermal and bronchial keratinocytes can be different according to the trigger. For example, bronchial keratinocytes can release cytokines in response to bisphenol A. Such an effect has not been illustrated in epidermal keratinocytes [114]. Interestingly, 22% of the retrieved articles did not involve keratinocytes. The culture conditions were not appropriate to obtaining conclusive data in 5% of the articles. Keratinocytes were, in some cases, present in a co-culture system with another cell-type or among a multilayer construct (3% and 4% of the articles, respectively); thus, the source of the reported secretions could not be related to a certain cell-type. Culturing the keratinocytes with another cell-type is important to address the effect of cell–cell interactions, and this affects the extracellular matrix development during cell differentiation [1]. The involvement of keratinocytes of non-human origin or abnormal keratinocytes represented 8% and 3% of the articles, respectively, and 12% of the research articles were not original, including review articles, case studies, and book chapters with one or more mentions of keratinocyte secretions.

### 3.2. Description of the Cell Secretions

**Figure 3:**
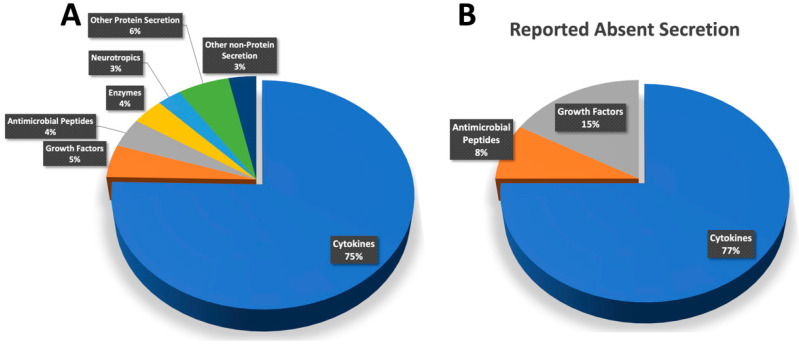
The reported present (**A**) and absent (**B**) secretions were mainly cytokines (75% and 75%, respectively), growth factors (6% and 17%, respectively), and antimicrobial peptides (5% and 8%, respectively).

Keratinocytes are the most common cells that belong to the neuroendocrine system in the skin [115]. For clarity, the reported secretions were classified into related groups: cytokines (75%), growth factors (5%), antimicrobial peptides (4%), enzymes (4%), neurotropic factors (3%), other proteins (6%), and non-protein secretions (3%) (Figure 3). The majority of the secretions belonged to the cytokine family, which could be due to the themes of the reporting studies. The latter investigated the effect of certain intermediaries on the production or inhibition of inflammatory mediators by keratinocytes or the effect of certain molecules to decrease such production, especially in inflammatory skin disorders. These inflammatory mediators are important in the wound-healing process, as they are associated with resistance to microbial infection while the skin is restoring its physiological barrier function upon wound closure. The production of cytokines by keratinocytes complements the physical barrier function of the skin with a chemically mediated action. Cytokine production is involved in the inflammatory process against infection, as well as in various skin disorders and hypersensitivity reactions [116]. The enzymatic production of keratinocytes helps cell migration through the degradation of the extracellular matrix, which is essential for wound repair. On the other hand, some of the articles reported the absence of the studied targets, which were mainly cytokines (77%), growth factors (15%), and antimicrobial peptides (8%).

The keratinocyte secretions in culture supernatant were ranked according to their reporting frequency in the selected studies (Table 1). The most common secretions were related to inflammation; interleukin (IL) 8 was mentioned in 39 studies, followed by IL-6 in 15 studies. Tumor necrosis factor was found in 15 studies and reported as absent in 3 studies. Other interleukins were also reported, including IL-1a (12 studies); IL-1β (7 studies); IL-12 (5 studies); IL-4 and IL-36 γ (3 studies); and IL-1ra (2 studies). Moreover, other immune mediators were also among the most highly reported secretions, such as C-X-C motif chemokine ligand 10 (CXCL10) in 9 studies; chemokine (C-C motif) ligand 5 (CCL5) in 5 studies; CCL20 (4 studies); CCL2, CCL22, and interferon gamma (IFNγ) in 3 studies; and others with lower reporting frequencies. These members of the cytokine family are known to induce each other in order to establish the appropriate inflammatory condition to combat infection through the interaction with Toll-like receptors. These receptors are present on the keratinocyte cell surface, as well as in the cytoplasm [116]. IL-8 has been extensively studied in skin disorders. This cytokine is released by epithelial cells in response to infection or injuries so as to attract the neutrophils that start the inflammatory process and enhance angiogenesis. The latter was achieved under the IL-8 paracrine effect on the dermal microvascular endothelial cells, which enhances the secretion of matrix metalloproteinases (MMP) 2 and 9. IL-8 has been correlated with the ability of melanoma cells to metastasize, and it has been blocked using anti-human IL-8 monoclonal antibodies in a preclinical trial [117]. Furthermore, the level of IL-8 in the stratum corneum can reflect the therapeutic efficiency of atopic dermatitis, and this has been correlated to the level of the serum inflammatory markers [118]. Interestingly, the role of cytokines may not be limited only to inflammatory processes, but also to enhancing cell differentiation. For example, the involvement of cytokines is crucial in guiding hematopoietic stem cells along their differentiation hierarchy [119]. In the skin, IL-1 can recruit a subset of lymphocytes that mobilize hair follicle stem cells and upregulate keratins 6 and 16, denoting actively migrating keratinocytes. TNF and IL-6, secretions reported in this review, as well as IL-17, are also involved in recruiting locally situated stem cells and progenitors for the wound reepithelization process through a similar mechanism [120]. Following burns, local IL-8 secretion steadily increased over the course of 24 h in an ex vivo human skin model [121]. IL-6 and IL-8 can induce angiogenesis at the wound’s edge, enhancing the repair process. Additionally, these interleukins can be induced from adipogenic-derived stem cells, present in the subcutaneous layer of the skin, under the effect of TNF [122]. The proinflammatory cytokines TNF, IL-1, IL-6, and interferon have a particular temporal expression in skin-wound repair. The release of these cytokines increases upon the invasion of pathological bacteria into the wound, which occurs during the first stage of wound repair. TNF and IL-6 are predominant in the second stage, when the granulation tissue is formed. In stage three, the same cytokines stimulate hair follicle regeneration and fibroblast proliferation. The importance of IL-6 has been confirmed in IL-6-deficient mice, as their wound healing was slower than that in normal mice. This effect was at least partially explained by the IL-6 effect on TGF beta [123]. Nevertheless, the concentration of these inflammatory mediators is crucial to supporting the healing process, as high levels of IL-1, IL-6, and TNF are usually present in chronic wounds [124]. A persistent, high level of TNF may prolong tissue inflammation and counteract keratinocyte and fibroblast proliferation, which can negatively affect the wound-repair process [123]. Similarly, several cytokines can be markedly upregulated by the reactive oxygen species produced in response to environmental challenges [125]. In the physiological range, reactive oxygen species are crucial for epidermal barrier integrity, hair development, and keratinocyte differentiation [126].

The second group of secretions are the antimicrobial peptides, including human β-defensin 2 (5 studies), neutrophil granule- and epithelial cell-derived cathelicidin, also known as LL-37 (3 studies), S100 and human β-defensin 3 (2 studies), and human β-defensin 1 (1 study). Antimicrobial peptides are considered to be a part of the natural defense mechanism of the skin. At least nine peptides can be secreted by keratinocytes, and this secretion can be induced in response to viral replication, which leads to an indirect antiviral effect [127]. The production of antimicrobial peptides can be upregulated through proinflammatory cytokines, such as IL-1β and TNF, which are reported in this study, as well as through IL-17 and IL-22 [116].

The growth-factors group is of particular interest for regenerative purposes. This group includes vascular endothelial growth factor (VEGF) and its endocrine-gland derivative, which were mentioned in four studies. Epidermal growth factor (EGF), granulocyte-macrophage colony-stimulating factor (GM-CSF), and transforming growth factor (TGF)-ß are reported in two studies, and there are single reports for nerve growth factor and TGF-α. Furthermore, there is a single report on the absence of platelet-derived growth factor (PDGF). Based on this analysis, the components responsible for the differentiation effect of keratinocyte culture supernatant on stem cells are difficult to speculate on, as most of the available studies have investigated keratinocyte secretions in the context of inflammation. The reported growth factors are important for keratinocyte migration during skin-wound repair, as well as the differentiation of locally situated epidermal stem cells into keratinocytes. Furthermore, different roles have been reported for these mediators in relation to skin stem cells. For example, the application of FGF2 into a skin wound has been associated with rapid healing in a mouse model. FGF2 has been found to induce keratinocyte morphology changes in spindle-shaped cells, a process similar to epithelial mesenchymal transition. Thus, the FGF2 pathway is crucial for the keratinocyte migration from the edge to the wound’s center [128]. The effect of FGF2 can be, at least partially, caused by the upregulation of the TGF- β cascade, which is also known as a driving factor in the differentiation of epidermal cells, as well as that of skin appendages [129]. Apart from its classic, angiogenic role, VEGF was shown to be involved in hair growth and wound repair. The decreased production of VEGF was associated with delayed wound-healing, as proven in diabetic and other chronic wounds. Furthermore, overproduction of VEGF and its receptor have been associated with skin disorders, such as psoriasis [130]. The association between VEGF, FGF1, FGF2, and CCL2 with the migration markers CXCR4 and MMP1 has confirmed the effect of paracrine secretion on local angiogenesis [122]. EGF has the well-established role of epidermal differentiation, as suggested by its name, as well as being a main component of several in vitro differentiation cocktails aimed at keratinocyte production [7]. Furthermore, EGF has an established role in wound-repair and is currently being investigated as a component of local applications for difficult-to-heal wounds [131].

TGF family members are constitutively expressed in the epidermal basal layer and are important for epidermal homeostasis, which is reflected by a high level of secretion during acute wound repair [132]. Members of the TGF pathways are involved in the development and maintenance of epidermal cells and skin appendages, such as hair, nails, and feathers, as well as in the angiogenesis process [124,129]. The paracrine effect of PDGF stimulates keratinocyte proliferation and migration, as well as the attraction of bone-marrow-derived stem cells, which enhances wound healing [133]. The absence of PDGF secretion in physiological conditions has been reported by a single study, highlighting the necessity of further studies in this area.

The enzyme group of keratinocyte secretions includes MMP1, MMP2, and MMP9 (2 studies), as well as MMP10 and kallikrein-related peptidase (1 study). The most commonly found MMP subtype in skin is MMP1, which cleaves the fibrillar collagen in the extracellular matrix. MMP2, MMP3, MMP9, MMP12, and MMP13 have mainly been reported to target elastic fibers [134]. The secretion of these MMPs provides access for keratinocyte migration. Metalloproteinases are usually expressed at the wound’s edge to support the mobility of keratinocytes, as well as to promote the secretion of TNF and VEGF to induce angiogenesis [135]. The action of metalloproteinase is regulated by the effect of the tissue inhibitors of metalloproteinases (TIMP), including TIMP2, as reported in this review. TIMP2 interacts generally with MMPs, but particularly with MMP2 and MMP9 in the skin [136]. On the other hand, kallikrein-related peptidase has an important role in the exfoliation process of aged keratinocytes [137]. The neurotropic group of secretions includes three reports on glutamate, as well as a single report on β-endorphin, artemin, and α-melanocyte-stimulating hormone (α-MSH). The role of keratinocytes in sensation not only involves the intraepidermal, free nerve endings, but also communication with theses endings through chemical mediators [138]. While glutamate acts as a neuromediator, artemin has a neuroprotective role, as well as a role in sensation perception [139]. β-endorphin has a local effect for pain management [140]. α-MSH serves locally to stimulate melanocytes for pigment production and regulate inflammation and extracellular matrix homeostasis through the receptor MC1R [141]. The other protein and non-protein groups reflect single reports of non-related secretions, including hyaluronan, which is an important component of the extracellular matrix. Hyaluronan has the property of retaining water, maintaining the flexibility of the skin. This polysaccharide influences cell migration, proliferation, and neovascularization through interaction with its receptors on the cell surface [142]. In a recent study, hyaluronan hydrogel was loaded with adipose-tissue-derived stem cells and applied to a burn-wound rat model. The study group demonstrated better healing proprieties, including wound closure and histomorphometry, in comparison to the control groups [143]. Different miRNAs have single reports, including miR-203, miR-675, and miR-3196, and they can be isolated from exosomes released by keratinocytes in a concentration parallel to the cell lysate. These miRNAs have been suggested as playing a role in melanogenesis [42,102]. Another important non-proteinous secretion of keratinocytes is sphingosine 1 phosphate, one of the lipid modulators that maintains skin integrity. This molecule has an important role in keratinocyte migration through the induction of MMP2 and MMP9, which have also been reported in this analysis, along with MMP1 and MMP10.

**Figure 4:**
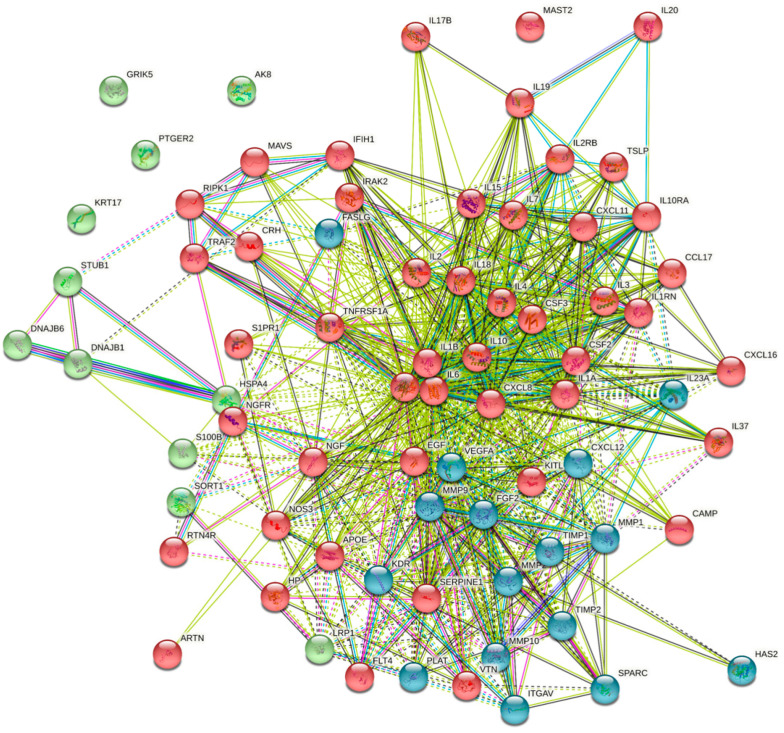
Clustering of the studied proteins shows three distinct clusters, with the growth factors on the border between the first and second clusters.

Analysis of the association between the secreted proteins, using k-means, shows three clusters (Figure 4). The biggest cluster contains the immune-related secretions, including the chemokines and cytokines, while the second cluster contains proteins related to wound healing. Interestingly, the growth factors are located at the interface between the two clusters, which suggests their central role in both processes. The biological processes related to the first cluster, according to GO-term, include the immune response and cytokine-mediated signaling pathway. The second cluster of biological processes includes extracellular matrix organization and disassembly, and the regulation of cell proliferation, differentiation, and migration, with an emphasis on keratinocytes and endothelial cells. Cluster 3 includes three nodes without significant enrichment. The protein association and functional enrichment analysis confirm the importance of keratinocyte secretions for immunological purposes as a first line of defense, in addition to their important role in enhancing wound-repair, through chemoattraction and differentiation induction on surrounding cells.

### 3.3. Stratification of Secretions According to Cell Type

The reported secretions were analyzed according to the cell source (Table 2). The secretions reported at least twice were included in this part of the analysis. Of the reported secretions, 52% were produced in primary keratinocytes, while 42% were produced in the HaCaT cell line. Out of the 29 secretions, 6 (21%) were reported as coming from 1 of the 2 cell types, while 23 secretions (79%) were reported as coming from both cell types although the included studies did not necessarily prove the ability of the other cell type to produce a particular secretion. For example, the use of HaCaT cells as a model for studying the secretory ability of β-defensin, IFNγ, IL-36γ, LL37, and IL-1ra may need prior validation, as these markers have been reported only in primary keratinocytes. On the other hand, EGF and GM-CSF secretion by primary keratinocytes should be further verified to match that previously reported for HaCaT cells. A few studies (2%) tested both primary keratinocytes and the HaCaT cell line to prove their results; accordingly, these studies were counted for each cell-type. The use of cell lines as models for in vitro experiments is a practical solution to compensate for the scarcity of primary cell donors, as well as to achieve higher proliferation rates and avoid donor-to-donor variability. The HaCat cell line has been used to model skin disorders and drug testing in vitro and in vivo through transplantation into murine models [144]. Unfortunately, cell lines do not necessarily reflect the physiological events that occur in primary cells or in vivo. In osteogenic differentiation, four cell lines were compared to primary-osteoblast- and bone-marrow-derived stem cells. The studied groups showed variability in cell viability and in alkaline phosphatase activity, an early marker of osteogenesis, upon being cultured under the same conditions [145]. In another study on the effect of glucocorticoids on the oral epithelium, the transcriptomic shift was not similar for the studied cell lines versus the primary isolated cells. Nevertheless, interesting genes were found among the commonly regulated genes in all the models [146]. Thus, further characterization of HaCaT cells is required to establish agreement with primary keratinocytes in terms of cell secretions.

## 4. Conclusions

The secretory functions of keratinocytes involve a wide range of biological processes which support skin functions. As skin represents the first line of defense against microbial invasion, its physical integrity is crucial. As it is subjected to friction and other physical forces, it is important to have an efficient repair system to maintain robustness. Multiple secretions are involved in skin regeneration, including a group of growth factors, as well as several cytokines. These secretions could be the main factor for the enhancement of stem cell differentiation into epidermal cells. There is a clear overlap between the functions of these secretions. At the same time, the absence of a global characterization of the keratinocyte secretome under physiological conditions illustrates the need for a comprehensive analysis of conditioned media components based on the results of this study. The detailed functional characterization of each of the included factors, as well as its different combinations, could lead the way to identifying the key inducers of stem cell differentiation into epidermal cells, and allow preparation of a chemically identified media for clinical use. Furthermore, the temporal effect of these factors should also be considered, which could lead to a multi-step, in vitro differentiation protocol. A significant limitation of this systematic review is the limited number of publications investigating the secreted growth factors. Thus, the clustering and functional enrichment analysis at this stage should be considered as preliminary. From another perspective, keratinocytes play an important role in secreting inflammatory mediators and antimicrobial peptides in order to combat infectious agents. Correlations between these secretions and different dermatopathological conditions may lead to the production of locally active and powerful compounds, such as topical treatments, as well as local biochemical tests for disease progression and monitoring of therapeutic effects. In addition, further characterization of keratinocytes’ secretory role during wound repair may lead to the development of other medicinal products that contain one or more synthetic alternatives to these secretions. In conclusion, this review highlights the opportunity for the formulation of an effective keratinocyte differentiation media based on keratinocyte secretion profiling. The latter has yet to be fully described and investigated. The multitude of keratinocyte secretions reported in this study may change our vision as to the nature of these cells.

## Figures and Tables

**Table 1:** The frequency of the reported presence or absence of secretions in cell culture supernatant.

n	Cell Secretion	Frequency of Presence	Frequency of Absence	n	Cell Secretion	Frequency of Presence	Frequency of Absence
**1**	IL-8	39		**41**	CXCL16	1	
**2**	IL-6	15	1	**42**	HSP70	1	
**3**	TNF	15	3	**43**	Human β-defensin 1	1	
**4**	IL-1a	12		**44**	IL-1 receptor	1	
**5**	Interferon inducible protein 10 (CXCL10)	9		**45**	IL-15	1	
**6**	IL-1β	7	2	**46**	IL-2	1	
**7**	RANTES (CCL5)	5		**47**	IL-20	1	
**8**	Human β-defensin 2	5	1	**48**	IL-23	1	
**9**	IL-12	5		**49**	IL-7	1	
**10**	CCL-20	4		**50**	IL-10	1	
**11**	CXCL1 (GRO-a)	4		**51**	IL-18	1	
**12**	Glutamate	3		**52**	IL-19	1	
**13**	CCL2/MCP1	3		**53**	IL-20	1	
**14**	FGF2	3	1	**54**	Fas ligand	1	
**15**	Hyaluronan	3		**55**	Granulocyte colony stimulating factor	1	
**16**	Interferon γ	3		**56**	Haptoglobin	1	
**17**	IL-4	3		**57**	IL-23p40	1	
**18**	IL-10	3		**58**	IL-3	1	
**19**	IL-36γ	3		**59**	Keratin 17	1	
**20**	LL37	3		**60**	Kallikrein-related peptidase	1	
**21**	Macrophage derived chemokine (CCL22)	3		**61**	Lympho–epithelial Kazal-type inhibitor	1	
**22**	Prostaglandin E2	3		**62**	Macrophage inflammatory protein (MIP)-1b	1	
**23**	VEGF	3		**63**	Macrophage inflammatory protein (MIP)-2	1	
**24**	EGF	2		**64**	Macrophage migration inhibitory factor	1	
**25**	GM-CSF	2		**65**	miR-203	1	
**26**	Human β-defensin 3	2		**66**	miR-675	1	
**27**	IL-1ra	2		**67**	miR-3196	1	
**28**	MMP1	2		**68**	MMP10	1	
**29**	MMP2	2		**69**	Nerve growth factor	1	
**30**	MMP9	2		**70**	Nitric Oxide	1	
**31**	S100	2		**71**	Serpin E1	1	
**32**	TGF-ß	2		**72**	Sphingosine 1 phosphate	1	1
**33**	Thymic stromal lymphopoietin	2	1	**73**	CCL17	1	
**34**	Adenylate kinase	1		**74**	Stem cell factor	1	
**35**	α-Melanocyte stimulating hormone	1		**75**	TGF-α	1	
**36**	Artemin	1		**76**	Tissue inhibitor of metalloproteinases 2	1	
**37**	β-endorphin	1		**77**	VEGF-EG	1	
**38**	Corticotropin-releasing hormone	1		**78**	p19/EBI3 heterodimeric cytokine complex		1
**39**	CXCL11	1		**79**	IL-37		1
**40**	CXCL12	1		**80**	PDGF		1

**Table 2:** The frequency of reported secretions in cell culture supernatant, according to the cell-type.

n	Cell Secretion	HaCaT	Primary Keratinocytes	Other Cell-Types	Total
**1**	IL-8	19	18	2	39
**2**	IL-6	10	4	1	15
**3**	TNF	6	8	1	15
**4**	IL-1a	3	8	1	12
**5**	CXCL10	2	7	0	9
**6**	IL-1β	4	3	0	7
**7**	human β-defensin 2	1	4	0	5
**8**	IL-12	2	2	1	5
**9**	RANTES (CCL5)	2	3	0	5
**10**	CCL-20	1	3	0	4
**11**	CXCL1 (GRO-a)	3	1	0	4
**12**	CCL2 /MCP1	1	2	0	3
**13**	CCL22	1	2	0	3
**14**	FGF2	2	1	0	3
**15**	Glutamate	1	2	0	3
**16**	Hyaluronan	2	1	0	3
**17**	IFNγ	0	2	1	3
**18**	IL-4	2	1	0	3
**19**	IL-36γ	0	3	0	3
**20**	LL37	0	3	0	3
**21**	Prostaglandin E2	1	2	0	3
**22**	VEGF	1	1	1	3
**23**	EGF	1	0	1	2
**24**	GM-CSF	1	0	1	2
**25**	IL-1ra	0	2	0	2
**26**	MMP1	1	1	0	2
**27**	MMP2	1	1	0	2
**28**	MMP9	1	1	0	2
**29**	TGF-ß	1	1	0	2

## Data Availability

Not applicable.
